# Basic needs in normative contexts

**DOI:** 10.1111/phc3.12732

**Published:** 2021-03-15

**Authors:** Thomas Pölzler

**Affiliations:** ^1^ Department of Philosophy University of Graz Graz Austria

## Abstract

In answering normative questions, researchers sometimes appeal to the concept of basic needs. Their guiding idea is that our first priority should be to ensure that everybody is able to meet these needs—to have enough in terms of food, water, shelter, and so on. This article provides an opinionated overview of basic needs in normative contexts. Any basic needs theory must answer three questions: (1) What are basic needs? (2) To what extent do basic needs generate reasons for action and how are these reasons to be understood? (3) How are basic needs and their satisfaction to be measured? I address these questions in turn. Then I also briefly discuss the theoretical potential of appealing to basic needs in normative contexts. It turns out that future research in this area could benefit from a higher degree of interdisciplinarity and methodological reflection, and that, generally speaking, basic needs theories are more promising than they have often been claimed to be.

## 1 INTRODUCTION

What do developed countries owe to people in developing countries? How much ought we to save for future generations? What grounds human rights? How to define sustainable development? In answering normative questions such as these researchers have sometimes appealed to the concept of basic needs (henceforth: BN). The guiding idea of these appeals is that our first priority should be to ensure that everybody is able to meet these needs—is able to have enough in terms of food, water, shelter, and whatever else is to be classified as a BN.

Needs have played a notable role in normative theorizing since its earliest days. In Western philosophy, for example, Aristotle discussed them in terms of necessities (Metaphysics 1981, 1015a–b15), and Marx famously argued that in communist societies, resources should be distributed “from each according to his ability, to each according to his needs” (1977: 569). *Basic* needs in particular had their heyday in the 1970 and 1980s, especially in the context of international development. Several proponents of early BN theories (such as Paul Streeten, Frances Stewart, and Norman Hicks) had connections to the World Bank, and developed and used these theories to measure poverty in developing countries.

In the last decades, BN theories have been gradually superseded by the so called capability approach, an alternative way of conceptualizing advantages and disadvantages that focuses more strongly on the value of people's freedoms. On closer consideration, however, the notions of “basic capabilities” (Sen, 1992) and “central capabilities” (Nussbaum, 2006) bear various similarities to BN (see fn. 26.); many proponents of capability theories occasionally fall back on BN language (e.g., Nussbaum, 2006, pp. 278–279); and recently, a growing number of researchers have also plausibly reemphasized BN's fruitfulness as distinct from capabilities (e.g., Gough, 2014; Meyer & Pölzler, forthcoming; Pinzani, 2013; Traub & Kittel, 2020). Understanding the role and potential of BN in normative contexts is thus still important.

This article provides an opinionated overview of BN in normative contexts. Any BN theory must answer three main questions: (1) What are BN? (2) To what extent do BN generate reasons for action and how are these reasons to be understood? (3) How are BN and their satisfaction to be measured? In what follows, I will address these questions in turn (Sections 1, 2, 3). Then I will also briefly discuss the theoretical potential of BN in normative contexts (Section 4). It will turn out that future research in this area could benefit from a higher degree of interdisciplinarity and methodological reflection, and that, generally speaking, BN theories are more promising than they have often been claimed to be. [Correction added on 05 May, 2021, after first online publication: Text error in the line “Then I will also…” has been corrected.]

## 2 THE NATURE OF BASIC NEEDS

The first question that any BN theory must answer is: What are BN? In academic contexts, it is not enough to just provide a list of purported BN (e.g., food, water, shelter, etc.). By themselves such lists neither explain why their items qualify as BN nor why satisfying BN is to be regarded as independently normatively relevant. BN theories rather need to be grounded in a substantive analysis which identifies necessary or characteristic features of the concept.

Perhaps the most famous analysis of BN was proposed by psychologist Abraham Maslow. Maslow postulated a “hierarchy of needs” (1943, 1954), according to which human motivation unfolds in distinct stages, first attending to unmet needs that are basic (Maslow characterized these BN as physiological and safety needs), and only then moving on to belongingless and love, esteem and self‐actualization. This psychological model of motivation has since been challenged (e.g., Tay and Diener 2011). Moreover, in normative contexts the term “need” is not meant to refer to certain inner states of beings (such as drives or urges) in the first place. Contrary to Maslow, normative researchers have rather been interested in needs in the sense of relations between beings and their environments (Doyal & Gough, 1991; Grix and McKibbin 2016).

BN *qua* relations have been analyzed based on different methodological assumptions. Philosophers have mostly started by attempting to capture ordinary people's intuitions. Only within constraints set by these intuitions, some of them have then “engineered” the concept to fit particular purposes.^1^ Braybrooke, for example, explains that “[w]hat […] needs are, met or unmet, is to be determined by inquiring what the concept of needs means to the people who have the concept” (1987: 39; see also Brock, 2005; Copp, 1998; Gasper, 2007). Social scientists, in contrast, have seemed more ready to regard “basic needs” as a technical term, and hence to define it freely to fit the particular purpose for which it is used (e.g., Hicks & Streeten, 1979; Stewart, 1985; Streeten & Burki, 1978).^2^

Personally, I am in favor of the philosophical (folk‐based) approach (see Pölzler, 2018). In most languages, the concepts of needs and BN are part of ordinary discourse. If an analysis does not account for this usage then it runs the risk of becoming incomprehensible, unacceptable or irrelevant to ordinary people (see Jackson, 1998; Machery, 2017; Miller, 1999). I even advocate for dedicated empirical research into ordinary people's conceptual intuitions about BN, in the sense in which such research has recently been conducted by experimental philosophers (see Knobe & Nichols, 2017).^3^ But of course, this methodological preference is controversial. In this section, I will thus mainly stick to reporting the broad outlines of the concept of BN, as they have been accepted by most philosophers and most social scientists.

BN are a subclass of needs, so it is natural to start our analysis by asking about the meaning of needs in general. Following Fletcher (2018), Reader (2006) and others, and reminiscent of Aristotle's analysis mentioned in the introduction, needs *qua* relations are probably best defined in modal terms, as a kind of necessities. To say that a person needs X means that X *must* be the case in order for some Y to be the case.^4^ This analysis is supported both by the similarity between “needs” and “must” propositions (e.g., “I need food” and “I must have food”) as well as by the fact that propositions such as “I must have food but I do not need food” are likely to strike many people as conceptually incoherent (see Fletcher, 2018).

Needs theorists have distinguished between various different kinds of needs. One of their main distinctions concerns the Y that need satisfaction is required for (Wiggins, 1998).^5^ An *instrumental* need is a need that must be satisfied in order to achieve some goal. My current need for a laptop is instrumental in this sense. If I did not have the goal of working on this article, then I would not need my laptop to achieve this goal. Relatedly, if I tell you that I need my laptop to work on this article I provide you with new information by ruling out other possible reasons that I could have for needing it (e.g., watching a YouTube video or reading the news; see Braybrooke, 1987; Thomson, 2005; Wiggins, 1998).

Other needs, in contrast, are *absolute*: they do *not* ontologically depend on anybody having any particular goal. Take my need for food. Even if I do not have the goal of eating—say, I am on a hunger strike—it is still the case that I *need* food. It would also be strange for you to ask “What do you need food for?” My answer to this question would not be explanatorily helpful.^6^ This is because with absolute needs the Y that their satisfaction is required for is conceptually predefined. We need the things that we need in an absolute sense (such as food) to avoid negative outcomes for our life or functioning; in particular, we need them to avoid being harmed (see again, e.g., Braybrooke, 1987; Thomson, 2005; Wiggins, 1998; for some researchers who have defined absolute or basic needs without reference to harm see Hassoun, 2008; Stewart, 1985; Wolf, 2009).^7^

Unsurprisingly, BN have been unequivocally defined as absolute (rather than instrumental) (e.g., Braybrooke, 1987; Reader, 2007; Wiggins, 1998). This raises the following questions: Are all absolute needs basic? If not, what distinguishes basic needs from other kinds of absolute needs? Researchers have used a variety of different labels to refer to absolute needs, basic needs and related but different kinds of needs, ranging from “fundamental” (Thomson, 2005), “vital” (Wiggins, 1998), “nonvolitional” (Frankfurt), “intrinsic” (Miller 1999) and “course‐of‐life” (Braybrooke, 1987) to “constitutive” (Grix & McKibbin, 2015) and “noncontingent” (Reader & Brock, 2004). This makes it difficult to understand and evaluate views on the relationship between absolute and basic needs in the sense defined above. It seems, however, that BN are dominantly taken to be a subclass of absolute needs that has two distinct features: the seriousness of the harm that is at issue, and the kind of necessity with which this harm arises.

Many researchers have noted that for any of a person's needs to count as basic, nonsatisfaction of this need must not only harm this person but must (at least in the long run) *seriously* harm her. Serious harm does not necessarily mean death. It is widely acknowledged that there are BN beyond mere survival, such as nonsurvival related aspects of health, clothing, or learning. But at least there needs to be some significant impairment to the “normal functioning” of the person (Braybrooke, 1987). In this vein, Doyal and Gough, for example, note that “basic needs are linked to the avoidance of serious harm” (1991: 50), and “if needs are not satisfied by an appropriate ‘satisfier’ then serious harm of some specified and objective kind will result" (1991: 39; see also, e.g., Feinberg 1973; Schuppert, 2013; and Thomson, 2005).^8^

The second distinguishing feature of BN concerns the particular way in which their satisfaction is necessary to avoid serious harm. This necessity is supposed to be nomological or quasi‐nomological. Most influentially, Wiggins suggested that BN are “entrenched” in the sense that their nonsatisfaction leads to harm because of “laws of nature, unalterable and invariable environmental facts, or facts about human constitution” (1998: 15). That is, a need can only count as basic if for humans there is no (or hardly any) way to avoid being (seriously) harmed when this need remains unsatisfied (e.g., Siebel & Schramme, 2020; Thomson, 2005).

The above basic clarifications still leave a number of important conceptual issues unsettled. For example, researchers have disagreed about how to understand the particular nature of the harm that the nonsatisfaction of BN necessitates. Some have argued that for a thing to qualify as a BN our deprivation of it must cause harm in the sense of impairing our autonomy (e.g., Copp, 1998; Doyal & Gough, 1991); others have (also) defined BN harm as a decrease in our (minimal or rational) agency (e.g., Brock, 2005; Copp, 1998; Schuppert 2013) or our capacity to fully perform the tasks that are associated with certain basic social roles (e.g., Braybrooke, 1987; Doyal & Gough, 1991; Miller, 2007).

Further important questions about the concept of BN concern its objectivity and universality. A large majority of researchers define BN as objective in the sense of being metaphysically independent not only from goals but from all kinds of mental states. For example, they hold that a person can have a BN for, say, education, even if she herself or the majority of the members of her culture does not believe that she has such a BN (e.g., Doyal & Gough, 1991; Pinzani, 2013; Thomson, 2005; but see Fraser, 1989). Relatedly, a majority of researchers also define BN as universal. On such definitions (almost) all individuals at (almost) all times and places necessarily have the same BN, whether they live today or 200 years from now, whether they reside in Japan or in Austria (see, e.g., Brock, 2005; Doyal & Gough, 1991; but see Hamilton, 2009; Wiggins, 1998).^9^

This is not the place to attempt to settle these controversial conceptual issues. Much will depend on the particular normative context at issue and on the methodology that one endorses for analyzing BN. Suffice it so say that one's stance on the nature of BN harm, the objectivity of BN, and the universality of BN significantly influences the content and plausibility of appeals to BN in normative contexts. This will also become apparent at several points in the following Sections.

## 3 THE NORMATIVITY OF BASIC NEEDS

As indicated in the introduction, BN can be and have been appealed to in all kinds of normative contexts, including international development, distributive justice, global justice, intergenerational justice, human rights, the social minimum, universal basic income, sustainable development and care ethics. This Section focuses on three challenges related to the normativity of BN that hold across all or most of these contexts: (1) establishing the independent normative relevance of BN, (2) specifying the bearers of obligations to meet these needs, and (3) explaining how to trade off BN satisfaction in non‐ideal conditions.

Any BN theory is motivated by the assumption that satisfying BN matters. More precisely, such theories assume that BN are independently normatively relevant, that is, the fact that one of a person's BN is unsatisfied by itself (irrespectively of any goals) generates a *pro tanto* reason to satisfy this need. For example, the fact that persons are starving by itself gives the state of Austria a *pro tanto* reason to enable these persons to have food (for discussion see, e.g., Copp, 1995; Reader, 2007).^10^ Is this assumption plausible?

Whether BN can be justifiably believed to be independently normatively relevant depends on how they are defined. In normative contexts, researchers should therefore deliberately strive to “engineer” the concept in a way that makes it likely that unmet BN indeed by themselves generate (strong) reasons for action. I take it that the widespread analysis suggested in Section 1 reflects such engineering efforts, especially in regards to its necessity and absoluteness components.

Since the nonsatisfaction of BN necessitates serious harm in view of laws of nature or presently highly invariable facts, people with unmet BN have no way to escape this harm (e.g., Frankfurt, 1984; Thomson, 2005; Wiggins, 1998). The harm is not contingent on anybody's goals either. This means that it is BN satisfaction as such—rather than these goals—that must be regarded as normatively relevant (Brock & Miller, 2019).^11^ Finally, some researchers have suggested that by defining BN as objective and universal the plausibility of their being independently normatively relevant increases even further (e.g., Doyal & Gough, 1991; Thomson, 2005).

However, while necessity, absoluteness, objectivity, and universality have desirable implications in terms of the above requirement, they also raise some intricate metaphysical issues. In particular, one may doubt that independently normatively relevant BN in the sense defined in Section 1 can even exist. This worry becomes particularly acute under the plausible assumption of a naturalistic metaphysics, according to which all entities or at least all moral entities are natural entities. To put it provocatively, how could a fact about what ought to be done belong to the same category of facts such as “Mount Everest is the highest mountain in the world” or “The average adult human brain weighs 1300–1400 g”?

Proponents of BN theories are hence challenged to vindicate and locate necessary, absolute, objective, universal, and independently normatively relevant BN in the natural world. This can be done, for example, by addressing familiar worries such as the one hinted at above, according to which BN *qua* independently normatively relevant, objective and natural entities would be “queer” in the sense of being very different from all other known kinds of entities (Mackie, 2011); or the worry that BN in this sense would not figure in our best explanations of any observations (Harman, 1977).

Suppose researchers meet this metaethical challenge, that is, they successfully establish that unsatisfied BN by themselves generate reasons to meet these needs (for a particularly elaborate attempt see Copp, 1995). The obvious next question to ask then is: Who are the agents to whom these reasons apply? In other words, who is it that ought to enable the satisfaction of BN; especially BN that we cannot meet ourselves?

In actual practice, some third‐party BN satisfaction occurs on a personal level. Care ethicists have thus stressed the reason‐generating power of caring relationships, such as between a mother and her child (Bubeck, 1995; Reader, 2007). Most often, however, BN have been appealed to in political normative contexts, with states as potential bearers of obligation. This makes good sense; for in many cases the (effective or morally appropriate) satisfaction of BN requires institutions or infrastructure that are simply beyond the reach of individuals. To illustrate, just by myself I cannot provide hip surgery to my grandma, manufacture a tractor or set up an unemployment insurance scheme.

In an ideal world, states would enable all people to fully meet all of their BN. Unfortunately, we find ourselves in conditions of moderate scarcity in which states often cannot or at least do not do so.^12^ This introduces the need for normative principles that guide trade‐offs, that is, that specify how obligations to satisfy some BN of some persons are to be weighed against (1) non‐BN related obligations and (2) obligations with regard to other BN or with regard to other persons (see Brock & Miller, 2019).

Any account of BN‐related trade‐offs will again crucially depend on general methodological decisions (see Siebel & Schramme, 2020). For example, proponents of needs theories have regarded normative principles as justified based on their own *a priori* normative intuitions (Wiggins, 1998), assumptions about the normative intuitions of ordinary people (Braybrooke, 1987; Copp 1995, 1998) and scientific data about these intuitions (Bauer et al., under review; Brock, 2005; Hassoun, 2009; Miller 2007, 2020). Personally, I favor drawing at least partly on scientific evidence. But again I will refrain from assuming this controversial methodology and instead confine myself to reporting on some widely accepted general claims.

In light of the independent normative relevance and harm‐related nature of BN, their satisfaction plausibly has priority over states’ non‐BN related obligations. First comes BN satisfaction; then these other obligations.^13^ This priority can take two forms: strong (other obligations can only apply once all BN of all persons are satisfied) or weak (even though it matters more to satisfy BN this satisfaction can be traded off under certain circumstances).^14^ In many normative contexts some version of the weak view seems more plausible; among others, because of what has been called the “black hole” problem (Huseby 2010; Shields 2016; for the label see Rendall, 2019).^15^

The black hole problem points to an implausible implication of the strong view. If BN satisfaction had strong priority then states would have to enable any citizen to satisfy her BN even if this BN satisfaction was extremely expensive (as with, say, some rare medical conditions). However, it seems that under some circumstances the opportunity costs of such an obligation could simply become too high to be justifiable. Rather than spending millions on meeting some particular BN of one particular patient states may redirect part of this money to increase the non‐BN related well‐being of many more people to a very large extent (e.g., by building a new art museum; e.g., Copp, 1998; Miller, 2020).

As stated above, BN theories also need to specify how enabling the satisfaction of some BN of some persons is to be weighed against enabling the satisfaction of other BN or other persons. In this process of weighing BN among themselves the “black hole” problem resurfaces. Should states always prioritize meeting the BN of those who are most deprived in BN satisfaction? If doing so is extremely costly—think, for example, of a terminally ill person whose life could be prolonged by expensive medical treatments—then again there seems to be a point at which it is permissible to limit expenditures and rather enable a much larger amount of BN satisfaction in many less needy people (e.g., by building a new wastewater system; see Copp, 1998; Wolf, 2009).^16^

What exactly, then, should weighting functions of particular BN obligations and (1) non‐BN related obligations and (2) other BN obligations look like? Many researchers accept that factors such as the absolute level of a person's deprivation, the amount of BN that can be satisfied and the number of people whose BN can be satisfied should be give some weight (for arguments in favor of some of these factors see Crisp, 2002; Meyer, 2009; Hassoun, 2009). But this may not yet be the end of the story. It might also be argued that states should take into account people's age (after a certain “normal” lifespan claims to BN satisfaction decrease; Copp, 1998), people's responsibility for their BN deprivation (the more responsible the weaker their claims; Segall 2014), and so on (for discussion of some of these further potential factors see, e.g., Copp, 1998; Miller, 1999).

In the end, BN‐based distribution under conditions of moderate scarcity may be a more complex normative matter than it has sometimes made out to be. It is not clear that any simple principle or combination of principles will be able to do sufficient justice to the multifaced normative intuitions that researchers (and ordinary people) have about such distributions (see Miller, 2020).

## 4 THE MEASUREMENT OF BASIC NEEDS

To explore the implications of BN theories for actual policies their proponents must identify BN, measure their satisfaction and specify ways of satisfying particular BN. These challenges will be addressed in the present Section. In terms of academic disciplines, they will shift our focus from philosophy to economics (or the social sciences more broadly).

BN‐related debates in philosophy and economics have so far lacked in integration, with only some notable exceptions (e.g., Doyal & Gough, 1991; Gough, 2017; Traub & Kittel, 2020). If we take philosophical considerations seriously—and we should!—then the only way to identify people's BN is to apply a well‐founded definition of BN to the actual world. For example, given the definition provided in Section 1, proponents of BN theories must investigate which things are necessary (given laws of nature or highly invariant facts) to avoid serious harm (in a sense to be specified) independently of the goals of individuals.

The result of any such application will be a list of BN involving abstractly described items such as food, water, shelter, clothing, and so on. On the assumption of the widespread method of reflective equilibrium (Daniels, 1979; Rawls, 1999)^17^, this list will not only be an implication of its underlying definition but can also be regarded as a test case for it. In many normative contexts, for example, a rather short list of BN will be helpful, that is, a list that only specifies requirements for a *minimally decent* life (e.g., Meyer & Pölzler, forthcoming; Miller, 2007). Thus, if a list turns out to be too expansive, then this might be taken as a reason to revise the list's underlying definition rather than to accept that the things entailed by the definition really are BN.

In any case, as BN are appealed to in a number of different normative contexts, it is unlikely that there will ever be one single “true” list of BN. The content of such lists will rather vary with these contexts. For example, researchers will end up with a list of BN that states must meet as a matter of ordinary (domestic intragenerational) distributive justice, a different list of BN that specifies their intergenerational duties of justice, a list that is most relevant to justifying human rights, and so on (for similar claims with regard to capabilities see Robeyns, 2005).

How can the satisfaction of any purported BN be measured? This largely depends on whether BN are regarded as objective or subjective. Subjective BN would most adequately be investigated by inquiring into people's views about BN (for more on such investigations see below). If BN are objective, in contrast, this means that both individuals and whole cultures can be mistaken in identifying their BN. Like the diabetic who thinks that she needs sugar but in fact needs insulin, the members of a culture may think that they don’t need, say, education even though they do need it. On this (plausible) latter assumption, BN satisfaction is thus to be primarily measured by objective social indicators (Doyal & Gough, 1991; Pinzani, 2013; Thomson, 2005).

The choice of adequate social indicators has been a matter of continued debate among BN theorists.^18^ Consider the contexts of international development and global justice. In these contexts, some researchers have argued that health is such a central BN, and life expectancy at birth is such a reliable indicator for health, that life expectancy at birth validly reflects BN satisfaction in general (Hicks & Streeten, 1979; Stewart, 1985). Other researchers have taken certain scores on the Human Development Index as a proxy of BN satisfaction, which, besides life expectancy, also includes mean and expected years of schooling as well as Gross National Income per capita (Williges et al., under review).^19^ Yet other researchers have suggested various existing indicators for each particular BN and have made proposals for which and how new indicators ought to be operationalized (Doyal & Gough, 1991).^20^

As lists and measures of BN are sensitive to the normative context at issue, and also to counteract charges of paternalism (see Section 4), I take it that it is recommendable for BN theorists to communicate information about them in cautious and respectful ways. In particular, researchers should try to avoid the impression that these lists and measures are definitive. Any lists and measures as well as their philosophical foundations need to be put forth for broad discussion not only by researchers but also by the general public (Stewart, 2006). Moreover, by contributing new objective evidence of the kinds outlined above it must always be possible to initiate revisions. (In these respects, as in many others, BN theorists can draw inspiration from how certain proponents of capability theories have framed their lists and measures of capabilities; see, e.g., Nussbaum, 2006; Robeyns, 2005.)

A crucial distinction in discussions about BN is between BN on the one hand and their satisfiers on the other hand, that is, those resources (objects, activities, relationships etc.) that have the potential to satisfy a particular BN (Max‐Neef, 1991). It is plausible that BN themselves are objective and universal. However, their satisfiers are clearly subjective and relative. Think, for example, of all the different ways in which people can and do satisfy their BN for food: Austrians eat Wiener Schnitzel; South Koreans have Bulgogi; Ethiopians prefer Addisu Gebeya (see, e.g., Brock, 2005; Doyal & Gough, 1991).

In light of BN satisfiers’ subjectivity and relativity, the question of what particular satisfiers states ought to provide in what particular ways can only be answered on the basis of investigations into people's attitudes. Among others, these investigations can take the form of social scientific studies (e.g., Costanza et al., 2007; McGregor et al., 2009; Pinzani & Rego, 2019) or of participatory processes, that is, processes that attempt to actively involve all members of a community in a decision‐making process, such as local workshops or citizen juries (e.g., Gough, 2017; Stewart, 1985, 2006; Streeten 1984).

But not all preferences need to be accommodated. Very likely, there are moral boundaries as to how states can be required to enable BN satisfaction. First, states seem to have a right to promote reasonably effective BN satisfaction (even if citizens would prefer a caviar‐only diet states do not have an obligation to provide it). Second, some researchers have plausibly argued that for reasons of self‐respect and autonomy^21^, wherever possible states ought not to *provide* but rather only *enable* BN satisfaction. For example, they ought not to hand out satisfiers (e.g., food packages) but rather create or maintain institutions which enable persons to meet their BN themselves, through their own efforts (e.g., through employment; Copp, 1998).

## 5 THE POTENTIAL OF BASIC NEEDS

In the past three Sections, I explained how proponents of BN theories must specify the nature, normativity and measurement of BN. Many of the challenges along this way are serious. Still, I am optimistic that with regard to most of them significant progress lies ahead, especially once investigations into BN will have become more interdisciplinary and methodologically reflective.

That said, even with the best possible BN theories at hand, for any particular normative question one might still ask: Are we really justified in appealing to BN in our answer to this question? For example, should we really make international development aid dependent on the level of BN deprivation in different countries (rather than on some other way of measuring well‐being)?

Assessing BN theories is essentially a comparative task: we should accept these theories if and only if they are better justified than alternative theories that fulfill the same theoretical function. In international development and other distributive contexts, for example, appeals to BN have mainly faced competition from views according to which well‐being is to be measured by the extent to which people's preferences are satisfied, people possess certain kinds of impersonal resources (such as income and wealth) or people have certain capabilities, that is, freedoms to do or be certain things.^22^ To support BN theories, one would hence have to show that on balance BN are more adequate in guiding distributions than any of these alternatives.

This is not the place to provide a full analysis of whether states should distribute goods according to people's BN, preferences, impersonal resources or capabilities. Space also does not permit comparing BN to alternative theories that have been proposed in other normative contexts. Instead, I will here limit myself to addressing three general criticisms that have been levelled at BN in almost all normative contexts in which they have been appealed to; especially at appeals to objective and universal BN. According to these criticisms, BN theories are problematic in that they are (1) paternalistic, (2) materialistic, and (3) passivity‐promoting. I will argue that these criticisms can be met in plausible ways (for further discussion see Brock, 2013; Gough, 2014; Fardell, 2020; Miller, 1999; Meyer & Pölzler, forthcoming; Reader, 2006; Wiggins, 2005).

The most prevalent and important objection to BN theories is that they are paternalistic. In the last Section, I suggested that on objectivist and universalist definitions, BN and their satisfaction are to be determined by objective scientific investigations. The results of these investigations can then be extrapolated across populations. For example, that members of Western cultures have a BN for education would imply the presence of this BN in members of non‐Western cultures as well. On the basis of such (objectivist and universalist) BN theories, states may thus end up providing people with satisfiers for BN that these people themselves regard as already being sufficiently satisfied or that these citizens do not even accept as BN. This indeed looks like a paradigm case of paternalism. In the words of Sen, it looks as if proponents of BN theories make the mistake of treating human beings “just as patients who have needs that require catering” but not “as agents who can think and act” (2013: 8; see also Alkire, 2002; Sen, 1984).

There is no point in denying that objectivist and universalist BN theories have an inbuilt tendency toward paternalism. However, as I have already touched upon in the last Section, this paternalistic tendency can be mitigated to a certain extent. Even if people are not free to decide that they do not have a BN for education or that this BN is already fully satisfied, they should at least be regarded as free (within certain moral limits) to choose *how* their BN are satisfied (education may rightfully take a very different form for Yanomami than for Chinese). I also explained how BN satisfaction might not be enabled by providing bundles of goods but by creating or maintaining institutions that allow people to meet their BN themselves (e.g., a system of free higher education). Finally, I suggested that any lists and measures of BN always need to be open for discussion and revision in light of relevant evidence.

Here are three further reasons for believing that BN theories can incorporate sufficient respect for people's autonomy (see Meyer & Pölzler, forthcoming).^23^ First, rather trivially, on these theories states are only required to *enable* people to meet their BN. This means that people are still free to be ascetics, illiterates, etc.; no adult is ever forced to eat, to read a book, etc.^24^ Second, a certain degree of autonomy may itself plausibly be regarded as a BN. This further limits the permissibility of autonomy‐decreasing state interferences (e.g., Brock, 2005; Doyal & Gough, 1991; Streeten, 1979). And third, for many BN theorists, a concern for autonomy is actually the ultimate reason why they attribute so much weight to alleviating deprivations of food, water, shelter, etc. in the first place (see Section 1). If humans are deprived of these things they are typically unable to fully develop and rationally pursue their own conception of the good life, as too much of their attention is focused on eliminating this deprivation (see Copp, 1995, 1998; Doyal & Gough, 1991).^25^

According to a second common objection against BN theories, these theories are “materialistic”. By this attribution, critics most often mean that such theories put too much emphasis on the material means of achieving well‐being rather than on well‐being itself—a problem that Sen famously termed “commodity fetishism” (1984: 510; see also Fisher, 1990), and that has also been raised with regard to impersonal resources as a measure of well‐being. For example, income by itself is not part of what it is to be well off; it only happens to be something that can be used to increase well‐being. To ground distributive decisions in considerations about income would therefore be to ground them in the wrong kind of thing.

However, as an objection against BN this again does not seem too convincing (see Meyer & Pölzler, forthcoming). On all plausible specifications of the harm of BN deprivation, the satisfaction of these needs ultimately only matters for nonmaterial reasons, for example, as just mentioned, because it enables persons to realize the (nonmaterial) value of autonomy. Moreover, humans can plausibly be attributed not only BN for food, water, and so on, but also for a number of nonmaterial goods. Even some of the earliest lists of BN already included education (Drewnowski 1979; Hicks & Streeten, 1979; Streeten & Burki, 1978), and more recent lists also include items like autonomy, self‐respect, or companionship (e.g., Copp, 1998; Doyal & Gough, 1991).

Finally, it has sometimes been objected that the concept of BN fosters dependency, and hence passivity on the side of those who receive BN satisfiers. Sen, for example, argues that when it comes to measuring well‐being, capabilities are preferable to BN because “‘[n]eeds’ is a more passive concept than ‘capability’ […] the perspective of positive freedom links naturally with capabilities (what can the person do?) rather than with the fulfillment of their needs (what can be done for the person?)” (1984: 215; see also Sen, 2009).

BN can indeed be understood as a form of dependency (see fn. 4). However, dependency in the relevant sense—dependency on our natural and social environment—is not bad (just as it is not good); it is simply a fact of life that every organism has to cope with (Reader, 2006). Critics’ attempts to deny or downplay human dependency could, on the contrary, be seen as approximating some form of hubris. BN theories would only be problematic if they fostered dependency *on the state*. As pointed out in the last Section, this can but perhaps need not be the case. Providing goods may not be the most preferable way of enabling BN satisfaction; state institutions could rather “empower” people by allowing them to achieve this satisfaction through their own efforts, thereby preventing direct dependency on the state, and the passivity that comes along with it (see Section 3).

## 6 CONCLUSION

This article explained essential elements of BN theories in normative contexts. I began by endorsing a widespread analysis according to which a thing is a BN if and only if it is necessary to avoid serious harm, independently of the goals of any agent (Section 1). Then I argued that it is plausible that BN are independently normatively relevant; that states are the main bearers of obligations to meet these needs; and that BN satisfaction (of the most needy) should have priority but not absolute priority (Section 2). Finally, I suggested that the methods of identifying and measuring BN satisfaction depend on whether these needs are defined as objective or subjective; and that citizens should at the very least have a say about the particular satisfiers of their BN (Section 3).

As mentioned in the introduction, in recent decades, BN theories have mostly been superseded by the capability approach. Section 4 defended BN theories against three general objections that have mainly been pressed by proponents of this approach, namely that BN theories are paternalistic, materialistic and passivity‐promoting. I argued that in certain forms appeals to BN may withstand these objections. This of course falls far short of establishing BN as superior to capabilities (or any other concept that has the same theoretical function). It should also be reemphasized that some versions of capability theories, such as the one by Nussbaum (see fn. 22), are in fact very similar to BN theories.^26^ Still, I hope that this article at least demonstrated that appeals to BN in normative contexts warrant further research.

To make progress on BN theories, two strategies appear to be particularly promising. First, proponents ought to reflect and improve on their general methods of philosophical justification, especially when it comes to analyzing the concept of BN and arguing in favor of principles for weighing BN satisfaction. My own (controversial) view is that in both of these regards researchers should initiate, collaborate in or at least draw on scientific research (such as psychological research on ordinary people's intuitions about BN). This already leads over to my second recommendation, which is a plea for increased interdisciplinarity. Besides the suggestion just made, this plea is especially targeted at the relation between philosophers and economists, who have so far often worked side by side rather than together, when it comes to BN.

Personally, I find a “first things first” attitude very appealing in many normative contexts. Before we go on arguing about the details of this or that complex and ambitious theory, we should be distinctly aware that people ought at least to be able to meet their BN; we should try to understand this claim and embrace it in our actions and policies.

## ENDNOTES

^1^
This latter component of conceptual analysis has recently been in particular stressed by proponents of so called “conceptual engineering” (Cappelen, 2018).

^2^
Even some philosophers agree that at least what it means for needs to be “basic” does not have to conform to folk intuitions (e.g., Wiggins, 1998). For a helpful general introduction to definition and measurement in the social sciences see Cartwright & Runhardt, 2014.

^3^
I have recently co‐developed an experimental design for measuring ordinary speakers’ intuitions about the concept of BN myself (Pölzler & Hannikainen, in preparation). Studies that are based on this design will be conducted soon.

^4^
The special kind of necessity that is expressed by needs propositions in normative contexts may be described as a form of dependency of organisms on their natural and social environment. To say that I need a thing is to say that I must have it in the sense that I depend on it in order for something to be the case, for example, to avoid harm (Wiggins, 1998). This connection between needs and dependencies has in particular been stressed by researchers in the tradition of care ethics (e.g., Dodds, 2013; Engster, 2007).

^5^
As will be mentioned below, the relation of the absolute/instrumental distinction to the basic/non‐basic discintion has remained somewhat unclear. Fletcher (2018) has recently criticized the absolute/instrumental distinction on two grounds. First, it is unclear whether the distinction is supposed to be between two kinds of needs, two concepts of needs or two senses of the same concept of needs. Second, he has argued that the distinction is non‐exhaustive, that is, there are needs which are neither instrumental nor absolute.

^6^
Here is another reason for believing that there is a kind of needs that is ontologically independent from goals, proposed by McLeod (2014). If all needs were instrumental (i.e., dependent on goals) then it would not make sense to ascribe needs to beings that lack goals. But it does seem to make sense to ascribe needs to beings that lack goals. For example, we say that a plant needs water or that a hamster needs a companion. As an aside: It is striking how widely proponents of BN theories in normative contexts have so far ignored questions about the BN of non‐human beings. For two of only few reasonably detailed considerations about how to expand the circle of BN concern to such beings see Attfield (1995), Reader (2007).

^7^
The proportion of discussants who have defended analyses of absolute or basic needs that do not refer to the notion of harm is rather small. To elaborate on the references provided above, Hassoun (2008) has suggested that a person has a need for X if and only if X enables the person to live a minimally good human life (understood as a life i.e. better than a life i.e. not worth living, but not as good as a good life); Stewart (1985) claims that a person has a BN for X if and only if X contributes to a full life; and according to Wolf (2009), a person has a BN for X if and only if X is necessary for the person to understand and exercise her rights and liberties.

^8^
Wiggins (1998), as one of only very few needs theorists, explicitly denies that the harm that arises from the nonsatisfaction of BN must be serious.

^9^
That basic needs are objective may seem to entail that they are also universal, that is, that all beings at all times and places have the same BN. Yet, while there is indeed a close connection between these claims, it is not one of entailment. Suppose the statement “P has a basic need for X” is made true by something objective—say, as is done here, by facts about what necessarily harms people. If our above definition of “necessarily” is combined with certain definitions of “harm” then it is still possible that these facts vary from person to person or, more plausibly, from culture to culture. For example, due to fundamental differences in language, upbringing, etc. people from different cultures may unavoidably develop different psychological traits that give rise to different psychological BN. For more on the distinction between the moral objectivism/subjectivism and universalism/relativism debate see Joyce, 2015.

^10^
A *pro tanto* reason to do X is a reason that only reflects one aspect of X and hence need not apply if all things are considered. Such reasons can be defeated by *pro tanto* reasons to not do X.

^11^
This has most famously been disputed by Barry (1965). According to him, BN are instrumental and what actually matters with regard to their satisfaction are hence only the goals for which the needed things are required (not the BN themselves).

^12^
Sometimes states are simply unable to fully meet the BN of all citizens. It might be disputed that this holds often. For example, Wolf (2009: 362) claims that “we have more than enough stuff to meet the basic needs of everyone, and needs provision is shockingly inexpensive.” However, as a matter of fact, even when states are able to fully meet the BN of all citizens (and even if they operate under conditions of little scarcity) they often do not do so, but rather prefer to devote resources to other matters.

^13^
In fact, in some contexts some researchers have suggested that enabling the satisfaction of BN is not only our first but our only obligation; see, for example, Miller, 2007 for our obligations towards people in other countries and Meyer & Roser, 2009 for our obligations towards future generations.

^14^
This question has most extensively been addressed in discussions about sufficiantarian „patterns“ of justice, that is, the views according to which justice requires ensuring that people have a sufficient level of well‐being (whether this level is defined in terms of BN, preferences, impersonal resources, capabilities or some different way). See, for example, Huseby, 2010; Shields, 2016.

^15^
This problem has also gone under other names. Brock and Miller (2019), for example, refer to it as the “bottomless pit” problem; Copp (1998) discusses it as the problem of “costly needs”. Another objection against strong priority views arises from considerations about risks. These views would implausibly require states to devote large amounts of resources to avoiding even very small risks of not satisfying BN (e.g., Meyer & Stelzer, 2018).

^16^
Similarly, at the level of kinds of BN (rather than persons), it seems permissible and probably required for states to limit expenditures for particular kinds of BN as well. For example, the resources that states could devote to fully meet citizens’ BN for health are basically infinite. But still they should care about other BN as well (see Brock & Miller, 2019; Miller, 2007).

^17^
Reflective equilibrium, which traces back to Rawls and was subsequently advanced by Daniels, is a method of justifying normative claims. It works by bringing coherence to (1) our considered normative judgments about particular cases, (2) the normative theories or principle that we believe govern these judgments, and, in so called “wide reflective equilibrium”, (3) non‐normative background theories. By refining our beliefs at these three levels a coherent system of beliefs (a state of reflective equilibrium) is supposed to emerge; and we are then epistemically justified in believing the elements of this system.

^18^
At least in the contexts of international development and global justice, many of these indicators have been shown to be closely correlated with each other (Stewart, 1985). So in the end one's choice may be of less consequence than it has often been assumed.

^19^
The most pressing problem for any composite index is how to aggregate the satisfaction of BN, that is, how to assign relative importance to different kinds of BN, such as the BN for shelter and the BN for education. For discussion see Hicks & Streeten, 1979; and for analogous discussions with regard to capabilities see Robeyns, 2005.

^20^
For example, for food and water Doyal and Gough (1991: 219) propose the following social indicators: (1) “Calorie consumption below FAO/WHO requirements,” (2) “Other nutrients consumption below requirements,” (3) “% lacking access to adequate safe water,” (4) “% suffering malnutrition/deficiency diseases,” (5) “% low birth weight babies”, (6) “% overweight/obese”. They suggest that 1, 3, 4 and 5 are already well‐established indicators, while with 2 and 6 data is incomplete but there are plausible ways of operationalization.

^21^
Self‐respect and autonomy may themselves be thought of as BN or even as the ultimate goal of BN satisfaction (see Section 1).

^22^
Under the heading of the capability approach, different theories have been developed. Sen (1992, 1999) at some places suggests that distributions should consider any capabilities that people value (though at other places he limits these capabilities to those that people have reason to value; and he also introduces a notion of „basic capabilities“). Nussbaum (2006), in contrast, clearly identifies ten „central capabilities“ that, in her view, states ought to promote: (1) life, (2) bodily health, (3) bodily integrity, (4) senses, imagination and thought, (5) emotions, (6) practical reason, (7) affiliation, (8) other species, (9) play, and (10) control over one's environment.

^23^
Another promising way of mitigating the paternalism worry questions what proponents of this worry would deem to be a “sufficient” level of autonomy. Some BN theorists have argued that critics exaggerate or even absolutize the value of free choice — they engage in what Reader (2006: 344) calls “choice‐ or freedom‐fetishism” (see also Brock & Miller, 2019).

^24^
In stressing this freedom BN theories move closer to the capability approach (which is centrally about individuals’ freedom to choose functionings).

^25^
BN theories’ tendency towards paternalism arises from their objectivity and universality. However, at the same time these potential features of the concept of BN also have advantages. Objectivist definitions shield BN theories from the so called “adaptive preference problem” (Elster, 1983). Universalist definitions ensure that we can have more certainty and knowledge about what we owe to people who are far way in space and time than we could otherwise have. For more details on these two advantages see, for example, Brock, 2005; Meyer and Pölzler forthcoming; Page, 2007.

^26^
For discussions of the differences and relative advantages/disadvantages between BN and central capabilities theories see, for example, Alkire, 2006; Gough, 2014; Fardell, 2020; Reader, 2006.
